# A case of gastrointestinal stromal tumor with spontaneous rupture in the greater omentum

**DOI:** 10.1186/1477-7800-5-19

**Published:** 2008-07-29

**Authors:** Norihiro Yoshimura, Hirotaka Ohara, Katsuyuki Miyabe, Tessin Ban, Hitoshi Sano, Itaru Naitoh, Kazuki Hayashi, Tomoaki Ando, Takahiro Nakazawa, Takashi Joh

**Affiliations:** 1 Department of Gastroenterology, Gifu Prefectural Tajimi Hospital, 5-161 Maehatacho, Tajimi, Gifu, 507-8522, Japan; 2 Department of Gastroenterology and Metabolism, Nagoya City University Graduate School of Medical Sciences, 1 Kawasumi, Mizuho, Nagoya, Aichi, 467-8601, Japan

## Abstract

**Background:**

Although GIST generally occurs in the digestive tract, such as the stomach, and small and small intestine primarily, Omental GIST tumours are very rare.

**Case Presentation:**

A 63-year-old male patient, who recognized an abdominal tumor 1 year before admission, had a slight expansion of the tumor, reduction of the body and malaise, was consulted to our hospital. Abdominal CT and MRI revealed a cystic lesion of 26 cm in diameter with a clear boundary from immediately below the interseptum to the pelvic cavity, and imaged the septum and cystic wall. We considered that the patient had a cystic tumor in the abdomen, of which the primary lesion was unknown, and scheduled surgery. The patient unfortunately deteriorated with shock and sudden pain in the abdomen. Wediagnosed tumor rapture, and emergency surgery was performed. The tumor, weighing 3,600 g, was mostly cystic, and filled with sanguinous fluid and clot. Histologically, the tumor was composed of spindle cells, and was positive for c-KIT (CD117), slightly positive for alpha-smooth muscle actin (SMA), and S-100 protein positive. Based on these findings, the tumor was diagnosed as GIST primarily occurring in the greater omentum.

**Conclusion:**

We experienced a rare case of GIST which originated from the greater omentum. Recently, the prognosis of GIST has been improved since the treatment with Imatinib.

It is necessary to consider the diagnosis of GIST on encountering a mass in the greater omentum.

## Background

Tumours occurring in the greater omentum are rare, and diagnosis of such lesions is difficult. Tumours with the immunohistochemical characteristics of gastrointestinal stromal tumor (GIST), of which the primary lesion is in the greater omentum, have recently been reported. Generally, GIST occurs in the digestive tract, and the incidence of primary GIST lesions in the greater omentum has been reported to be less than 1%. We describe a patient with massive GIST occurring primarily in the greater omentum, which subsequently ruptured spontaneously during the observation period, necessitating emergency surgery.

## Case presentation

A 63-year-old male, who recognized an abdominal mass 1 year before this admission, presented with a slight expansion of the tumor, weight loss of 5 kg in 3 months, and malaise. A massive non-tender abdominal tumor was palpated. Haematological examination found anaemia and high levels of CRP and LDH, while the levels of CEA, CA19-9, and AFP were within the normal ranges.

**Figure 1:**
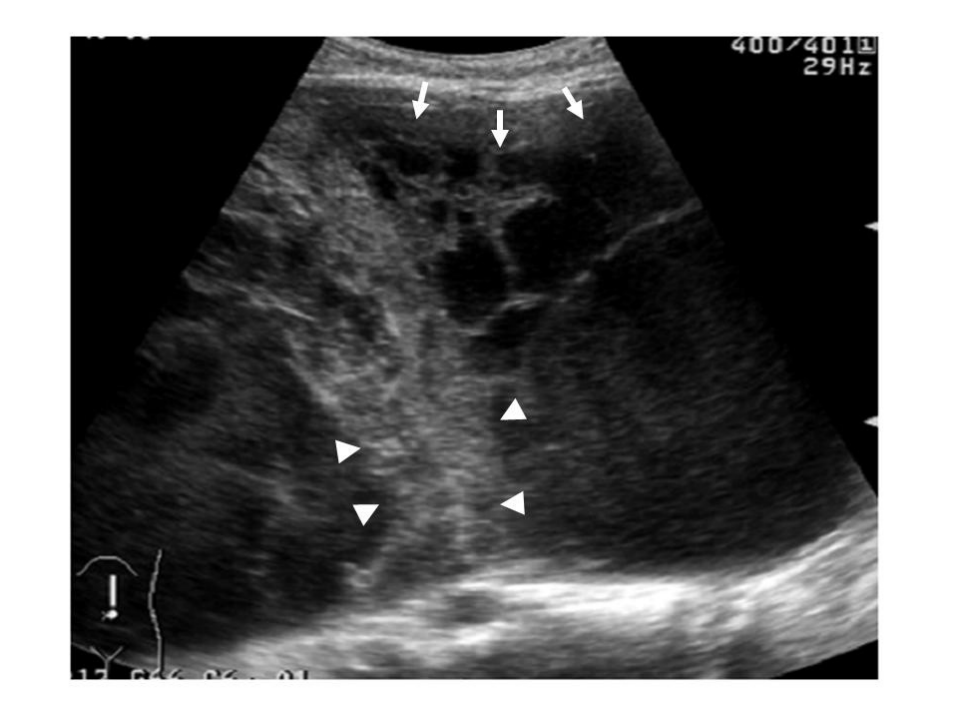
**US demonstrated peripheral microcyst clumps (arrows) and solid areas (arrowheads) in the abdominal massive cystic cavity**.

**Figure 2:**
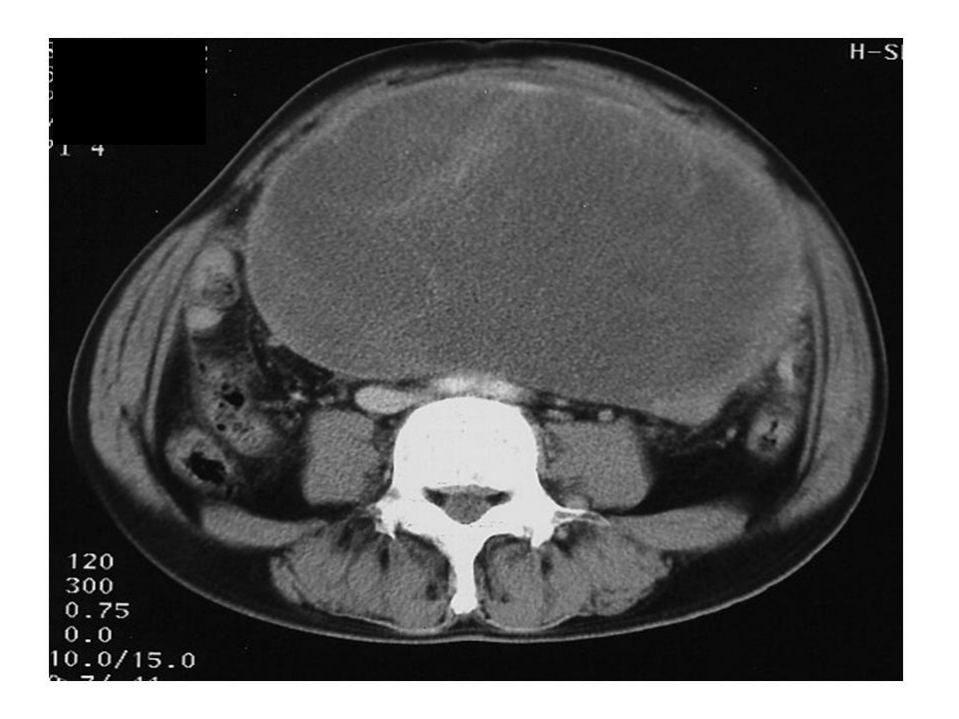
**Contrast-enhanced CT revealed a large tumor in the entire abdomen and showed septum in the cystic tumor**.

**Figure 3:**
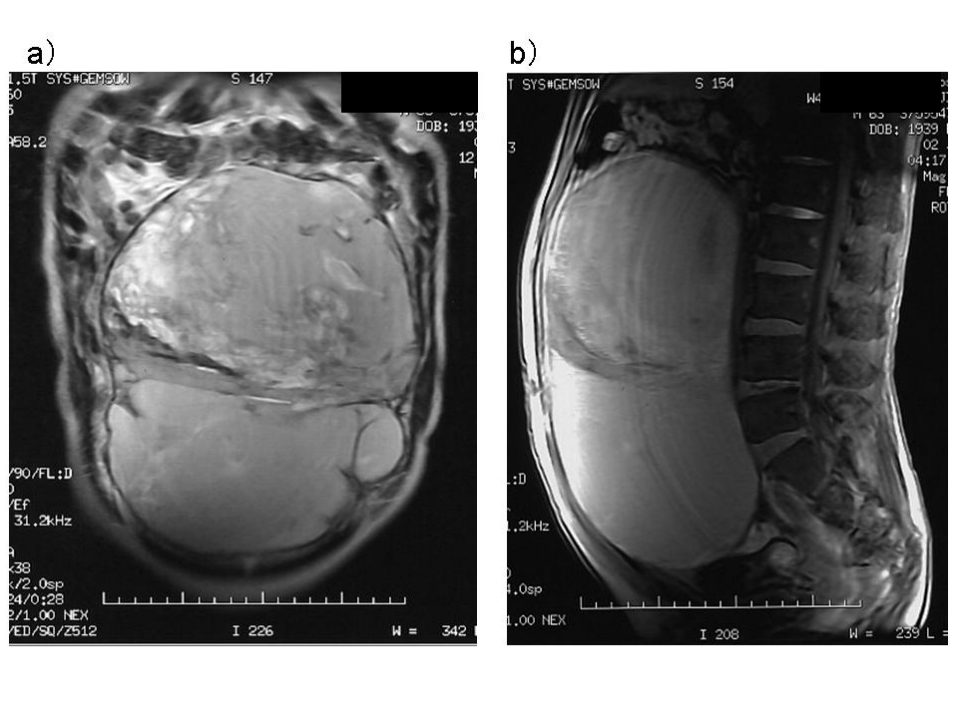
**MRI detected a thick septum in the tumor center, which divided the tumor into the upper and lower regions**. T2-weighted imaging showed coexistence of areas with high and low signal intensity. (a) coronal T2-weight imaging (b) sagittal T1-weight imaging.

**Figure 4:**
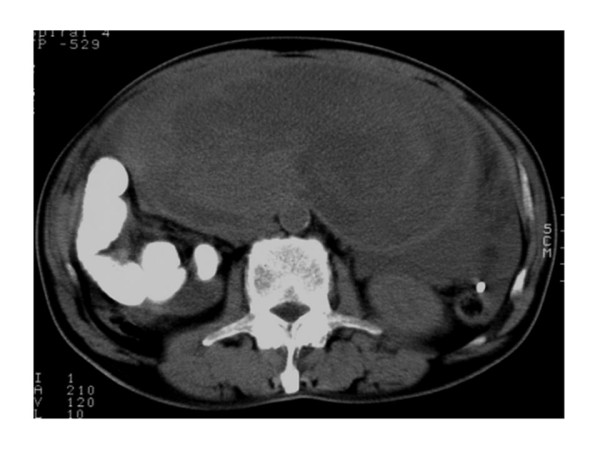
**Emergency CT revealed reduction of the tumor and retention of ascites**. Arrows: past contrast medium used gastroenteric examinations.

Abdominal ultrasonography found microcyst clumps and some solid areas in the periphery of the lesion. (Fig. 1) Abdominal CT (computed tomography) revealed a cystic lesion of 26 cm in diameter with a clear boundary from immediately below the interseptum to the pelvic cavity, and imaged the septum and cystic wall. (Fig. 2) No ascites were detected. MRI was performed (magnetic resonance imaging) and revealed a thick septum in the tumor center, which divided the tumor into the upper and lower regions. T1- and T2-weighted imaging showed slightly high signal intensity within the tumor, and T2-weighted imaging showed coexistence of areas with high and low signal intensity. (Fig. 3) There was no continuity between the tumor and the surrounding organs. Angiography revealed no enhancement of the tumor but exclusion of blood vessels by the tumor. The findings by endoscopy of the upper digestive tract, contrast of the small intestine, and enema were normal. We considered that the patient had a cystic tumor in the abdomen, of which the primary lesion was unknown, and scheduled surgery. Unfortunately, the patient developed shock and abdominal pain before the scheduled day of surgery. The tumour became unclear by palpation, and CT revealed reduction of the tumor and development of ascites. (Fig. 4)

Hematological examination showed no aggravation of anemia.

**Figure 5:**
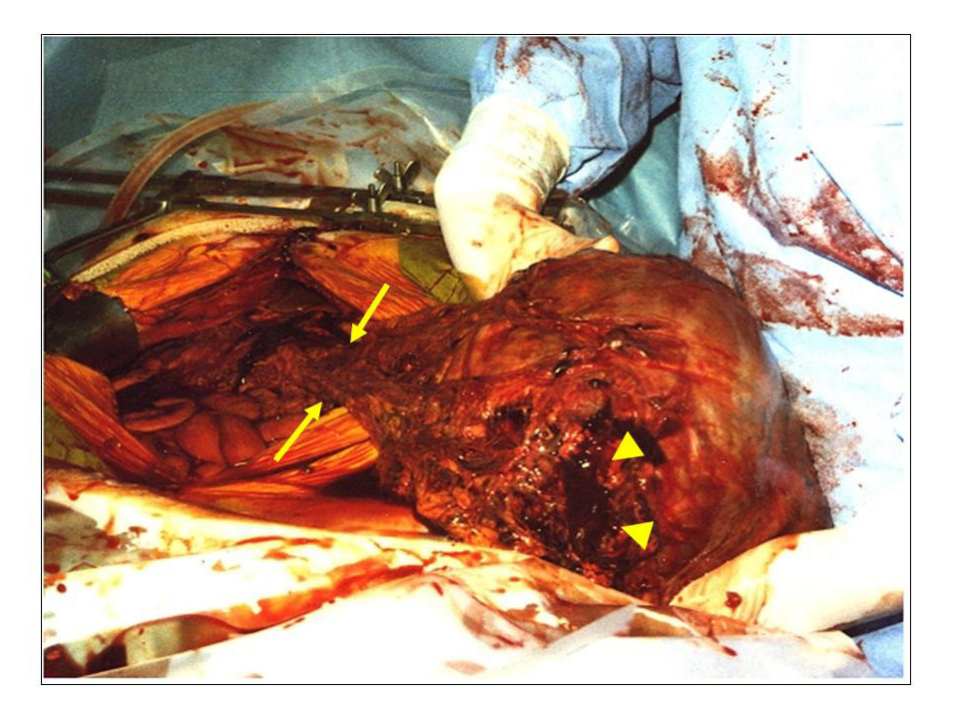
**Intraoperative findings showed tumor localized in the greater omentum**. (arrows). A rupture about 4.5-cm long was observed on the right side of the tumor. (arrowheads).

**Figure 6:**
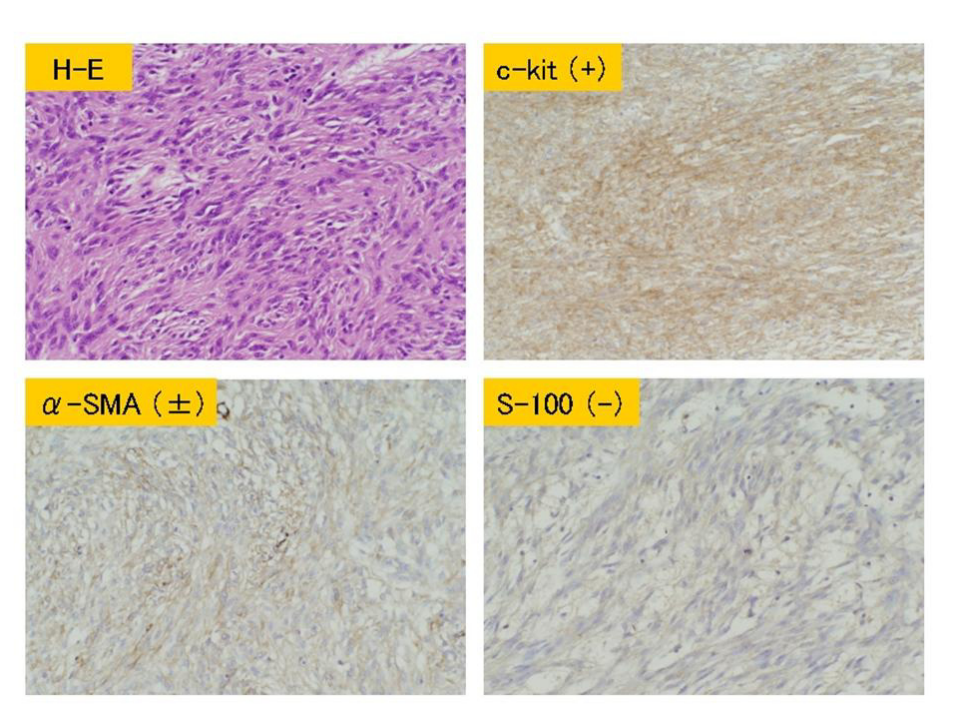
**Microscopic findings of the resected specimen**. Histologically, the tumor mainly consisted of spindle cells, which showed fascicular growth. Immunostaining demonstrated c-kit positive, partial α-SMA positive, and S-100 protein negative.

We considered that the cystic tumor had ruptured, and performed emergency surgery on the day. There was about 2,000 ml of sanguinous ascites in the abdominal cavity, and a rupture about 4.5-cm long was observed on the right side of the tumor. (Fig. 5) The tumor occurred primarily in the greater omentum, with no adhesion to the surrounding organs. A large number of peritoneal buds with a size of 5–10 mm were observed on the abdominal wall and in the small intestine. Total excision of the tumor, including the greater omentum, was performed. The tumor, weighing 3,600 g, was mostly cystic, and filled with sanguinous fluid and clot. Solid regions were observed partially on the cystic wall. Microscopically, the tumor mainly consisted of spindle cells, which showed fascicular growth. Immunostaining demonstrated c-kit positive, partial α-SMA positive, and S-100 protein negative. (Fig. 6) Based on these findings, the tumor was diagnosed as GIST primarily occurring in the greater omentum. Postoperatively, the patient was undergoing chemotherapy with STI571 for the treatment of the abdominal buds, and was alive as of 13 months after surgery.

## Discussion

Generally, GIST occurs primarily in the digestive tract, such as the stomach, and small and large intestine, and the incidence of the primary GIST lesion in the greater omentum is very unusual[1]. Among mesenchymal tumors on the digestive tract wall, KIT-expressing tumors are regarded as GIST, which are considered to be derived from the interstitial cells of Cajal cells[2].

It has been reported that GIST in the mesentery and greater omentum, structures which lack ICCs, are derived from mesenchymal cells that are less differentiated than ICCs[3], ICC precursors straying into the abdominal cavity[4], or KIT-positive cells similar to ICCs immediately below mesothelial cells in the greater omentum[5]. However, the precise aetiology remains to be clarified.

A single patient with spontaneous rupture of GIST in the greater omentum during the observation period has been reported by Shingu et al [6]. They considered that hemorrhage and cystic changes are likely to occur in the greater omentum because it is mainly composed of sparse membrane structures with abundant blood flow, resulting in spontaneous rupture. In our patient, nothing unusual with regard to pathological significance was observed in the ruptured region, and some load on the tumor in addition to its development and changes in the cysts may have caused the rupture. Our patient is the second reported case of spontaneous rupture of GIST in the greater omentum. One should consider GIST as a differenfial diagnosis on encountering a tumour in the greater omentum.

## Competing interests

The authors declare that they have no competing interests.

## Authors' contributions

NY Documented and prepared the draft. HO: Contributed towards revising the manuscript critically and has given final approval for the version to be published, KM Literature search and edited part of the manuscript. TB Edited part of the manuscript and interpreted the radiological images. HS Literature search and edited part of the manuscript. IN Revision of bibliography and edited part of the manuscript. HK Examined the surgical specimen and provided the histological photographic slides. TA Contributed towards conception, design, analysis and interpretation of data. TN Contributed towards conception, acquisition of data and preparation of the draft. TJ Contributed towards revising the final manuscript critically. All authors read and approved the final manuscript.
